# Assessment of Antimicrobial Properties of Phenolic Acid Extracts from Grain Infected with Fungi from the Genus *Fusarium*

**DOI:** 10.3390/molecules27051741

**Published:** 2022-03-07

**Authors:** Anna Przybylska-Balcerek, Tomasz Szablewski, Renata Cegielska-Radziejewska, Tomasz Góral, Danuta Kurasiak-Popowska, Kinga Stuper-Szablewska

**Affiliations:** 1 Department of Chemistry, Faculty of Forestry and Wood Technology, Poznań University of Life Sciences, 60-628 Poznań, Poland; kinga.stuper@up.poznan.pl; 2 Department of Food Quality and Safety Management, Faculty of Food Science and Nutrition, Poznań University of Life Sciences, 60-624 Poznań, Poland; tomasz.szablewski@up.poznan.pl (T.S.); renata.cegielska-radziejewska@up.poznan.pl (R.C.-R.); 3 Department of Applied Biology, Plant Breeding and Acclimation Institute—National Research Institute, Radzików, 05-870 Błonie, Poland; t.goral@ihar.edu.pl; 4 Department of Genetics and Plant Breeding, Faculty of Agronomy, Horticulture and Bioengineering Poznan University of Life Sciences, 60-632 Poznań, Poland; danuta.kurasiak-popowska@up.poznan.pl

**Keywords:** pathogens, fungi, *Fusarium* spp., bacteria, cereal grains, antibacterial, antifungal, antimicrobial

## Abstract

Problems related with biological contamination of plant origin raw materials have a considerable effect on prevention systems at each stage of food production. Concerning the antimicrobial action of phenolic acids, studies were undertaken to investigate antibacterial properties against bacterial strains of *Escherichia coli* (EC), *Pseudomonas fluorescence* (PF), *Micrococcus luteus* (ML) and *Proteus mirabilis* (PM), as well as antifungal properties targeting microscopic fungi *Fusarium* spp., extracts of phenolic compounds coming from inoculated grain from various genotypes of cereals. This study evaluated the antimicrobial action of phenolic acids extracts obtained from both naturally infested and inoculated with microorganisms. For this purpose a total of 24 cereal cultivars were selected, including 9 winter and 15 spring cultivars. The analyses showed a bactericidal effect in the case of 4 extracts against *Micrococcus luteus* (ML), 14 extracts against *Pseudomonas fluorescence* (PF), 17 extracts against *Escherichia coli* (EC) as well as 16 extracts against *Proteus mirabilis* (PM). It was found that 3 out of the 24 extracts showed no antibacterial activity. In turn, fungicidal action was observed in the case of 17 extracts against *Fusarium culmorum* (FC) (NIV), 16 extracts against FC (3AcDON), 12 extracts against *Fusarium graminearum* (FG) (3AcDON), while 12 other extracts showed antifungal action against FG (NIV) and 19 extracts against *Fusarium langsethiae* (FL). Based on the conducted analyses it was found that grain of small-grained cereals exposed to fungal infection is a source of bioactive compounds exhibiting antimicrobial properties. It was observed that the qualitative and quantitative profiles of polyphenols vary depending on the cereal cultivar. This extracts may be used to develop an antimicrobial preparation applicable in organic farming.

## 1. Introduction

In Central Europe the dominant mycoflora in grain of small-grained cereals consists of microscopic fungi, primarily the genus *Fusarium* spp. [1,2,3,4] They are considered to be the most pathogenic and phytotoxic microorganisms [1,2]. These fungi produce secondary metabolites, jointly referred to as mycotoxins, in view of their harmful effect on plants, animals and humans [5,6]. 

The global problem related to biological contamination of raw materials and food products of plant and animal origin has a considerable impact on prevention systems at each stage of food production. In the case of plant food the stage determining product contamination is connected with plant vegetation. However, every stage of plant raw material processing may lead to contamination with pathogens. Taking into consideration consumer expectations, natural alternatives are being searched for both in plant protection and in the protection of products aiming at a reduction of chemical applications in agriculture. Plant extracts exhibit antimicrobial properties. To date such studies have been dedicated to herbs, spices and fruit. Extensive investigations on the subject have concerned primarily bacteria and yeast (Table 1).

A significant difficulty is related to the lack of reports in the available literature on the effect of phenolic compounds extracts on pathogenic, toxigenic strains of microscopic fungi of the *Fusarium* genus. Due to their cell structure as well as advanced mechanisms of resistance towards various types of antimicrobial agents these microorganisms pose particularly serious problems as food contaminants. An incentive for this study was provided by interesting findings described in publications. It was stated that during mass pathogen infestation in cereal grain we can observe enhanced biosynthesis of phenolic compounds, which provide the first line of defence against pathogens. In such a situation plant cells initiate defence mechanisms in response to stress [26]. In a non-enzymatic system plant cells produce compounds, which reduce the negative effects of ROS. These compounds are then generated in such concentrations that with no external factors being present (e.g., pesticides) they scavenge free radicals and restore homeostasis in infected plant cells. These compounds exhibit antioxidant action. Antioxidant activity is exerted e.g., by vitamins, macro- and microelements, as well as plant origin substances such as polyphenols [5,27,28,29,30].

The group of compounds exhibiting antioxidant properties include, among others, also phenolic acids. Studies conducted to date indicate that phenolic compounds are inhibitors of free radical reactions [7,30,31]. In terms of the structure of the basic carbon skeleton phenolic acids are derivatives of benzoic acid (gallic, *p*-hydroxybenzoic, protocatechuic, syringic acids) or cinnamic (chlorogenic, ferulic, caffeic, *p*-coumaric, sinapic acids). In cereal grains, phenolic acids are found at slight concentrations depending on the species and grain maturity [30,32].

In view of the above the following research hypothesis was proposed: intensive polyphenol biosynthesis in grain induced by inoculation with microscopic fungi from the genus *Fusarium* leads to the generation of a complex of such compounds, which should exhibit antifungal activity. No such studies have been conducted to date. Thus after extracts were prepared they were tested initially on selected bacterial strains. In order to verify the above-mentioned hypothesis simultaneous analyses were performed using extracts from cereal grain, in which no fungal infection was induced. A total of 24 cereal cultivars were selected for analyses. Cereals were grown under identical cultivation conditions, in this way excluding the potential effect of other stressors, which could affect the level of polyphenols in grain.

Literature on the subject published to date presents practically no information on antimicrobial action of phenolic acids. In view of the above, the aim of the study was to assess antibacterial properties against bacterial strains *Escherichia coli* (EC), *Pseudomonas fluorescence* (PF), *Micrococcus luteus* (ML) and *Proteus mirabilis* (PM), as well as antifungal properties against microscopic fungi from the genus *Fusarium* spp., extracts of phenolic compounds from inoculated grain of various genotypes of cereals grown in Poland.

## 2. Materials and Methods

### 2.1. Experiment

**Figure 1:**
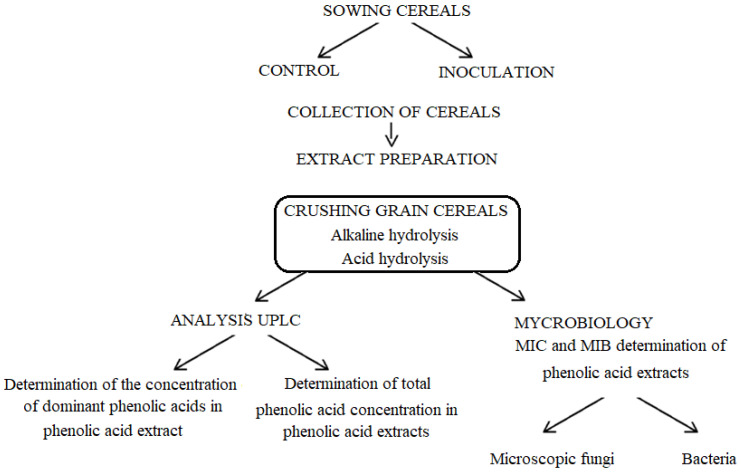
Course of experiments.

The experiment was planned according to the scheme presented in the diagram in Figure 1.

### 2.2. Field Experiments

In field experiments conducted in Poland in the years 2017–2019 at the Dłoń Agricultural Research Station Dłoń a total of 15 spring cereal genotypes and 9 winter cereal genotypes were tested (Table 2).

### 2.3. Inoculation of Cereals

Grain of the above-mentioned genotypes was sown in two replications (control, inoculated) in plots of 12 m^2^. The inoculant was a mixture of conidia from 5 of microscopic fungi from the genus *Fusarium*, i.e., *F.* *culmorum* (Wm. G. Sm.) Sacc. (2 isolates), *F. graminearum* Schwabe (2 isolates) and *F. langsethiae* Torp & Nirenberg (isolate 8051). Isolates of *F. culmorum* (KF 350) and *F. graminearum* (ZFR 119) belonged to chemotypes producing nivalenol (NIV), while isolates of *F. culmorum* (KF 846) and *F. graminearum* (ZFR 29) were chemotypes generating deoxynivalenol (DON) and 3-acetyldeoxynivalenol (3AcDON) [33,34].

Isolates were incubated on autoclaved wheat grain in glass flasks in the dark at a temperature of 20 °C for approx. 1 week and next irradiated with UV light (350 nm) for 12 h/24 h for approx. 3 weeks at 15 °C. Grain overgrown with mycelium with visible sporulation of *Fusarium* spp. Was dried and stored at 4 °C. On the day of inoculation the grain with mycelium and spores was soaked in water for approx. 2 h and next filtered to produce a spore suspension. The concentration of spore suspensions was determined using a Thoma cell counting chamber. The concentration was approx. 5 × 10^5^ spores/mL for *F. culmorum* and *F. graminearum*, while it was 10^6^ spores/mL for *F. langsethiae*. Suspensions were mixed at equal proportions [35,36,37].

Genotypes were inoculated at the stage of anthesis by spraying ears with a spore suspension, conducted for each treatment separately depending on the date of flowering. Ears were sprayed with a spore suspension at approx. 100 mL suspension per 1 m^2^. Inoculation was performed on each plot at the beginning of flowering and it was repeated approx. 3 days later at anthesis. At this phase cereals were most sensitive to ear infection by *Fusarium.* Inoculation was performed in evening hours, when relative humidity was increasing. During harvest 100 ears were collected manually from each plot. Ears were threshed using a laboratory thresher at low air flow in order to prevent losses of slightly infested kernels.

A total of 48 cereal grain samples (24 control samples and 24 samples after inoculation). Each grain sample weighed approx. 2 kg. Grain samples were analysed in 3 replications.

### 2.4. Preparation of Phenolic Extracts

Samples for analyses were 5 g. They were placed in sealed 250 mL round bottom flasks, where first alkaline and then acid hydrolysis was run. In order to run alkaline hydrolysis 25 mL distilled water and 100 mL 2M aqueous sodium hydroxide were added to the test flask. Tightly sealed test flasks were heated in a water bath at 95 °C for 30 min. After cooling (approx. 20 min) test flasks were neutralised with 50 mL of 6M aqueous hydrochloric acid solution (Ph = 2). Next, samples were cooled in water with ice. Phenolic acids were extracted from the organic phase using diethyl ether (2 × 50 mL). Formed ether extracts were continuously transferred to 120 mL vials. Next acid hydrolysis was run. For this purpose the aqueous phase was supplemented with 75 mL of 6M aqueous hydrochloric acid solution. Tightly sealed test flasks were heated in a water bath at 95 °C for 30 min. After being cooled in water with ice the samples were extracted with diethyl ether (2 × 50 mL). Produced ether extracts were continuously transferred to 120 mL vials, after which they were evaporated to dryness in a stream of nitrogen [29,38].

Prior to microbiological analysis the extracts obtained according to the above method were dissolved in 25 mL of water.

### 2.5. Chromatographic Analysis of Phenolic Acids

Samples for analyses were 0.20 g in weight. They were placed in sealed 17 mL culture test tubes, where first alkaline and then acid hydrolysis were run. In order to perform alkaline hydrolysis 1 mL of distilled water and 4 mL of 2M aqueous sodium hydroxide were added to test tubes. Tightly sealed test tubes were heated in a water bath at 95 °C for 30 min. After cooling (approx. 20 min) the test tubes was neutralised with 2 mL of 6 M aqueous hydrochloric acid solution (pH = 2). Next the samples were cooled in water with ice. Phenolic acids were extracted from the inorganic phase using diethyl ether (2 × 2 mL). Next acid hydrolysis was run. For this purpose the aqueous phase was supplemented with 3 mL of 6 M aqueous hydrochloric acid solution. Tightly sealed test tubes were heated in a water bath at 95 °C for 30 min. After being cooled in water with ice the samples were extracted with diethyl ether (2 × 2 mL). Produced ether extracts were continuously transferred to 8 mL vials, after which they were evaporated to dryness in a stream of nitrogen. Prior to analyses samples were dissolved in 1 mL methanol. Analyses were performed using an Aquity H class UPLC system equipped with a Waters Acquity PDA detector (Waters, USA). Chromatographic separation was performed on a Acquity UPLC^®^ BEH C18 column (100 mm × 2.1 mm, particle size 1.7 μm) (Waters, Ireland). The elution was carried out in gradient using the following mobile phase composition: A: acetonitrile with 0.1% formic acid, B: 0.1% aqueous formic acid mixture (pH = 2). Gradient changes during the analysis of phenolic compounds (A-0.1% solution, B-0.1% solution) [time [min]: A:B 0.0 min. 10:90; 2.0 min. 10:90; 15.0 min. 28:72; 22.0 min. 30:70; 22.1 min. 80:20; 24.0 min. 90:10; 24.1 min. 95:5; 26.0 min. 95:5; 35.0 min. 97:3; 35.1 min. 80:20; 45.0 min. 80:20; 55.0 min. 10:90; 60.0 min. 10:90]. Concentrations of phenolic acids were determined using an internal standard at the wavelength λ = 280 nm. Compounds were identified based on a comparison of retention times of the analysed peaks with the retention time of the standard and by adding a specific amount of the standard to the analysed samples and a repeated analysis. The detection level was 1 μg g^−1^. Retention times of assayed acids are as follows: gallic acid 8.85 min, vanillic acid 9.71 min, protocatechuic acid 12.23 min, vanillic acid 14.19 min, 4-hydroxybenzoic acid 19.46 min, chlorogenic acid 21.56 min, caffeic acid 26.19 min, syringic acid 28.05 min, p-coumaric acid 40.20 min, ferulic acid 46.20 min, sinapic acid 48.00 min and t-cinnamic acid 52.40 min, respectively. Recovery rates for the analysed phenolic acids were as follows: gallic acid 92 ± 4%, vanillic acid 79 ± 8%, protocatechuic acid 90 ± 4%, vanillic acid 88 ± 5%, 4-hydroxybenzoic acid 96 ± 3%, chlorogenic acid 92 ± 2%, caffeic acid 86 ± 6%, syringic acid 94 ± 3%, p-coumaric acid 89 ± 3%, ferulic acid 91 ± 4%, sinapic acid 94 ± 5% and t-cinnamic acid 97 ± 2% [29,30].

### 2.6. Analysis of Antimicrobial Properties

Microbial analyses were conducted on selected microscopic fungi from the genus *Fusarium*, i.e., *F. culmorum*—KF 350 producing NIV (FC NIV) and KF 846 producing 3AcDON (FC 3AcDON), *F. graminearum*—ZFR 119 producing NIV (FG NIV) and ZFR 29 producing 3AcDON (FG 3AcDON) and *F. langsethiae* (FL 8051), as well as bacteria—*Escherichia coli* (EC) PCM 2793, *Pseudomonas fluorescence* (PF) PCM 2123, *Micrococcus luteus* (ML) PCM 525 and *Proteus mirabilis* (PM) PCM 1361. Strains of *Fusarium* originated from the collection of Tomasz Góral from the Plant Breeding and Acclimatization Institute—National Research Institute in Radzików, while bacterial strains were obtained from the Polish Collection of Microorganisms, the Institute of Immunology and Experimental Therapy, the Polish Academy of Sciences in Wrocław.

Antimicrobial efficacy of analysed preparations was determined based on Minimum Lethal Concentration (MLC) and Minimum Inhibitory Concentration (MIC) applying the suspension cell assay. For this purpose a series of dilutions were prepared for the analysed extracts in sterile deionised water and they were introduced at 5 cm^3^ to test tubes containing 4 cm^3^ of respective medium and 1 cm^3^ suspension of the tested microorganism to that the final cell density was 10^7^/cm^3^. Samples were incubated for 72 h at 37 °C. Next cell growth and viability of tested microorganisms in successive test tubes by screening liquid bacterial cultures on solid TSA medium (Oxoid), while fungi were transferred onto MEA (Oxoid). After 48 h microbial growth was determined on Petri dishes. The evaluation was based on an organoleptic evaluation.

The lowest concentration of the preparation inhibiting microbial growth in liquid culture (with the microorganism growing on solid medium) specified the MIC value, while the lowest concentration of the preparation, at which the microorganism showed no growth on the solid medium showed the MLC value [39].

### 2.7. Statistical Analysis

**Figure 2:**
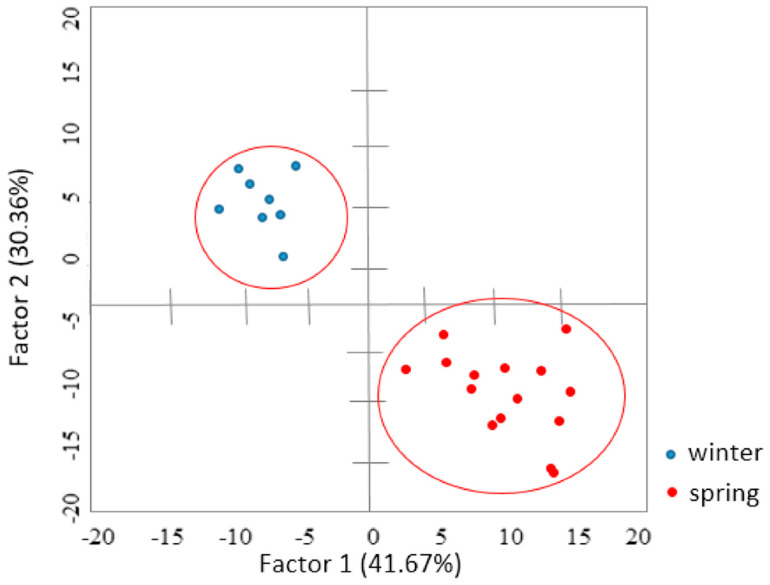
PCA analysis results, case projection on the plane for the entire population and all variables. Lambda Wilk < 0.001, *p* < 0.001. Ellipses are 95% confidence intervals.

Statistical analysis was performed in the Statistica ver. 13.1 software. Based on the results of chemical and microbiological analyzes, the PCA analysis was carried out and the Pearson correlation method at the confidence level of 0.95 (Figure 2).

## 3. Results

This study assessed antimicrobial effects of extracts of phenolic acids obtained from selected cultivars of small-grained cereals, both naturally infested and inoculated. For that purpose a total of 24 cultivars were selected, of which 9 were winter cereal cultivars and 15 were spring cereal cultivars. From 3 replications a bulk sample of 2 kg was collected, from which a sample of 100 g grain each was used in further analyses. Chemical analyses of phenolic acid concentrations were performed in 2 replications, with the means shown in Table 3.

Based on the PCA analysis covering all cases and all variables (concentration of phenolic compounds, MIC and MCB). Based on the discriminant analysis, it was found that ferulic acid (Lambda Wilk’s 0.3011; F_removal_ = 44.2188), naringenin (Lambda Wilk’s 0.1217; F_removal_ = 9.2233), and sinapic acid (Lambda Wilk’s 0.0927; F_removal_ = 12.7742), had the highest discriminatory power. The separation that was obtained indicates that the present compounds make it possible to separate the spring and winter cereal population. The classification matrix allowed, based on the concentration of ferulic acid and naringenin, to classify all analyzed samples in 100% into two groups: spring and winter crops.

The correlation analysis performed for all cases showed significant correlations between the MIC and the content of ferulic acid (r^2^ = 0.7034), naringenin (r^2^ = 0.6581), t-cinnamic acid (r^2^ = 0.7223) and sinapic acid (r^2^ = 0.6787).

The presence of pathogenic bacterial strains and microscopic fungi is one of the stressors, which as a result of metabolic processes contributes to the production of reactive oxygen species (ROS, reactive oxygen species) [5]. Concentrations of microscopic fungi from the genus *Fusarium* in naturally infested plant cells is frequently very low, although it is sufficient to pose a threat to cells contributing to disturbance of homeostasis. The crucial aspect is connected with the concentration of pathogenic microscopic fungi in plant cells. Under physiological conditions in cells a relative balance is maintained between the level of produced oxygen radicals and antioxidant activity. While cell infestation with a pathogen is slight, it may nevertheless lead to permanent changes followed by cell death, since there was no impulse to trigger natural defence mechanisms. In turn, during inoculation, when the concentration of pathogenic microscopic fungi is significantly higher and effects of oxidative stress are aggravated, as a consequence excess ROS is formed. At that time in response to stress plant cells trigger defence mechanisms [26]. In the case of a non-enzymatic system the compounds limiting negative effects of ROS are produced in plant cells. In these situations these compounds are produced in such concentrations that without the participation of external factors (e.g., pesticides) they scavenge free radicals and restore homeostasis in infected plant cells. These compounds, exhibiting antioxidant action, include e.g., polyphenols [5,28,29]. In view of the above based on the conducted analyses it was stated that all the extracts obtained from grain of the control cereals showed no antimicrobial effect on selected pathogenic microscopic fungi or bacteria. This is connected with the too low intensity of stressors, during which in plant cells no rapid increase in ROS levels occurred and thus antioxidant compounds were not produced. The concentration of phenolic acids in extracts was too low to exhibit antimicrobial activity.

### 3.1. Antibacterial Effects

Based on the conducted analyses it was stated that extracts of phenolic acids originating from inoculated grain have antibacterial and antifungal activity towards selected strains. None of the extracts obtained from grain of the control cereals without inoculation of *Fusarium* species at flowering showed antibacterial effect.

Experiments were conducted in order to verify antibacterial properties of phenolic extracts obtained from selected inoculated cultivars of small-grained cereals. For this purpose reference bacterial strains were selected, which are pathogenic not only to plants, but primarily to humans. Results showed antibacterial action of tested extracts of phenolic acids towards bacteria, i.e., *Escherichia coli* (EC), *Pseudomonas fluorescence* (PF), *Micrococcus luteus* (ML) and *Proteus mirabilis* (PM) (Table 3).

Among the 24 tested extracts all were found to exhibit bactericidal activity against ML bacteria. Extracts coming from grain of hybrid rye (Tur) and fodder barley (Harris) showed lowest activity against ML bacteria, with their MLC amounting to 0.07% and 0.05%, respectively. In turn, 2 other active preparations were obtained from grain of triticale (Palermo) and fodder barley (Argento), while their MLC amounted to 0.74 and 1.00%. The other 20 extracts showed bactericidal activity, but only at higher concentrations (Table 3). Based on analyses (Table 4) additionally it was stated that the above-mentioned 4 preparations exhibited bacteriostatic action at concentrations of 0.04–0.59%.

The conducted analyses showed antibacterial activity for all tested preparations also against PF bacteria. The lowest MLC (0.6–0.73%) and MIC (0.48–0.58%) were recorded for extracts coming from grain of wheat (Astoria), open pollinated rye (Agrikolo), fodder barley (Harris), malting barley (Irina) and triticale (Nagano). Other preparations obtained from grain of hybrid rye (Tur), fodder barley (Argento) and triticale (Milkaro) had MLC within the range of 0.9–1.0%, while their MIC values were 0.73–0.8%. Bactericidal and bacteriostatic effects were also observed for extracts from grain of triticale (Palermo, Milewo), malting barley (Nokia), hulless oat (Amant), wheat (Torka) and hulled oat (Nawigator). Their MLC values were over 1.0%, whereas MIC was lower amounting to 0.82–1.17%. In the case of extracts obtained from the other 10 cereal cultivars only bactericidal action against PF bacteria was recorded.

The next stage of the study analysed the activity of phenolic acid extracts towards EC and showed their bactericidal effect. The lowest MLC (0.6–0.73%) and MIC (0.48–0.58%) were found for extracts obtained from grain of wheat (Astoria), fodder barley (Harris), malting barley (Irina) and triticale (Nagano). Antibacterial activity against EC was also recorded for extracts from grain of wheat (KWS Ozon), open pollinated rye (Rostockie), fodder barley (Argento), triticale (Milkaro, Milewo), hulled oat (Bingo), from grain of triticale (Palermo, Dublet), rye (s74n05), hulless oat (Amant, Siwek) and wheat (Kandela, Torka). Their MLC was 0.84–1.46%, while MIC amounted to 0.67–1.17%, respectively. The other 7 extracts showed only bactericidal action towards EC.

The last of the 4 pathogenic bacteria to be analysed was PM. The highest antibacterial activity and thus the lowest MLC (0.6–0.74%) and MIC (0.48–0.59%) were recorded for 4 out of 24 tested samples. They were extracts coming from grain of fodder barley (Argento), malting barley (Irina), wheat (Astoria) and triticale (Nagano). Based on this analysis it was also found that MLC of two from the tested phenolic acid extracts exceeded 0.84, while the bacteriostatic effect was observed in the case of 12 other extracts and the other 8 preparations exhibited only bactericidal activity (Table 3, Table 4).

### 3.2. Antifungal Activity

None of the extracts obtained from grain of the control cereals without inoculation of *Fusarium* species at flowering showed antifungal effect.

In the last stage of this study experiments were conducted to verify antifungal properties of phenolic acid extracts obtained from selected inoculated cultivars of small-grained cereals. The results showed antifungal activity for most analysed phenolic acid extracts against *Fusarium* spp.: *Fusarium culmorum* (FC), *Fusarium graminearum* (FG) and *Fusarium langsethiae* (FL) (Table 4).

Extracts coming from grain of hybrid rye (Tur), triticale (Borowik) and wheat (Torka) exhibited the highest activity towards FC producing NIV, while their MLC was 0.25% and MIC was 0.20%. Among the 24 tested samples 3 had MLC of 0.50% and MIC of 0.40%. In turn, for 7 of all the analysed extracts MLC was 0.75% and MIC was 0.60%, respectively. Based on the results presented in Table 3 and Table 4 it was stated that extracts of phenolic acids coming from 7 other cultivars of tested cereals exhibited only fungicidal action towards this pathogen.

Another fungal pathogen included in the analyses was FC producing 3AcDON. Antifungal activity against this fungus was observed for phenolic acid extracts from grain of 3 cereal cultivars, i.e., open pollinated rye (Agrikolo) and triticale (Borowik and Nagano). MLC of these extracts in relation to this pathogen was 0.5%, while MIC amounted to 0.40%. Moreover, it was stated that among the 24 tested extracts 8 had MLC of 0.75% and MIC of 0.60%. In turn, in the case of 8 other extracts no inhibitory action was found, with only fungicidal effect observed towards FC producing 3AcDON.

Next analyses concerned the activity of phenolic acid extracts towards a microscopic fungus FG producing 3AcDON. Antifungal properties in this case were observed for phenolic acids extracted from grain of open pollinated rye (Rostockie, Agrikol) as well as triticale (Nagano). Their MLC amounted to 0.25%, while MIC was 0.20%. Among all the tested extracts 2 had MLC of 0.50% and thus MIC of 0.40%. In the 6 other preparations MLC amounted to 0.75%, while MIC was 0.60%. Moreover, it was observed that a half of all the extracts showed no inhibitory activity, whereas their fungicidal activity was recorded towards FG (3AcDON).

Analyses were also conducted on another pathogen FG producing NIV. The greatest activity against it was recorded for extracts of phenolic acids obtained from grain of wheat (Astoria), triticale (Dublet) and hulless oat (Siwek), with MLC amounting to 0.25% and MIC- to 0.20%, respectively. Based on the presented results it was stated that extracts of phenolic acids coming from 6 other cereal cultivars had MLC of 0.75% and MIC of 0.60%. In turn, those from 12 other tested cereal cultivars showed no inhibitory activity towards this pathogen a exhibited only fungicidal properties.

The last of the 5 tested fungal pathogens was FL. The highest antifungal activity and thus also the lowest MLC (0.25%) and MIC (0.20%) were recorded for 5 of the 24 samples. They were extracts from grain of open pollinated rye (Rostockie, Agrikolo), fodder barley (Argento), wheat (Kandela) and triticale (Nagano). The results also showed that MLC of 3 among all analysed extracts was 0.5% and their MIC was 0.40%, while 5 other extracts had MLC of 0.75% and MIC of 0.60%. It was also observed that 5 extracts showed no antifungal action towards this pathogen.

### 3.3. Synergistic Action of Phenolic Compounds

Based on these analyses it was observed that the qualitative and quantitative profiles of these low molecular antioxidants vary depending on the type and cultivar of the cereal (Table 5, Table 6). This study consisted in the preparation of extracts of phenolic compounds obtained from different cereal cultivars and it confirms that these compounds may exhibit synergism in their bactericidal and fungicidal action towards selected bacteria and *Fusarium* fungi. In many cases a stronger action was observed for a mixture of dominant polyphenols rather than individual compounds (Table 3, Table 4). Based on this study it was stated that in all the tested grain samples ferulic acid was the dominant phenolic acid. Due to the high content of this acid in grain of cereals it is ascribed antioxidant, antibacterial and antifungal properties in relation to the investigated pathogens. In turn, the antimicrobial activity of the extract increases when it is combined with sinapic and gallic acids, naringenin or quercetin [32,40].

## 4. Discussion

Phenolic acids belong to a numerous group of polyphenols with strong antioxidant properties, which are used in the prophylaxis of diseases, such as virus and bacterial infections as well as mycoses, while they may also be applied as an alternative to conventional pesticides. They exhibit diverse effects towards pathogenic organisms, either slowing down or completely inhibiting their growth. The currently available literature on the subject contains a vast body of data on the structure and antioxidant properties of these compounds. This analysis add information is presented on the antibacterial and antifungal action of phenolic acids. This study confirmed the antifungal activity towards selected fungi from the genus *Fusarium.* Literature on the subject reports that substances of natural origin, such as chlorogenic, ferulic and benzoic acids, may be effective fungicides against *Fusarium oxysporum* [41]. In the case of infestation with a pathogenic microscopic fungus *Sclerotinia sclerotiorum*, which causes rot, preparations containing chlorogenic and ferulic acids proved to be effective [42]. Other researchers in their studies showed antifungal activity of phenolic acids in terms of their toxicity towards *Fusarium graminearum*: chlorogenic acid < p-hydroxybenzoic acid < caffeic acid < syringic acid < p-coumaric acid < ferulic acid [42]. In other investigations the same scientists stated that phenolic acids exhibit a much higher antifungal activity against *F. culmorum* compared to other fungi from the genus *Fusarium*, which was also confirm in this study. In literature were observed an inhibitory effect of cinnamic, sinapic, caffeic, p-coumaric, chlorogenic and ferulic acids on the production of type B trichothecenes in the case of *F. graminearum* and *F. culmorum*, while derivatives of benzoic acid, excluding syringic acid, activated biosynthesis of mycotoxins. It was also found that inoculation of small-grained cereals with microscopic *Fusarium* fungi species stimulated production of phenolic acids in grain. This was confirmed by a study of Kulik et al. [43], who showed that microscopic fungi from the genus *Fusarium* contribute to more intensive generation of phenolic acids in plant cells.

Other scientific reports indicate that derivatives of benzoic acid exhibit antimicrobial activity both towards microscopic fungi and yeasts from the family *Candida*, e.g., *Candida albicans* at pH < 5.0. Similarly as in this study, Czechowska et al. in their research also observed that phenolic acids limit growth of microscopic fungi, i.e., *Fusarium* spp., *Aspergillus* spp. and *Penicillium* spp. In turn, benzoic acid in the presence of other polyphenols showed synergistic fungicidal action against *Cryptococcus neoformans* [5,44].

This study also verified antibacterial properties of preparations obtained from various cultivars of small-grained cereals. The analyses confirmed antibacterial action of phenolic acid extracts obtained from selected cereal cultivars in relation to bacteria, i.e., EC, PF, ML and PM. Literature on the subject presents only scarce reports related to the discussed properties of phenolic acids as growth inhibitors for bacteria from the genera *Yersinia*, *Bacillus*, *Corynebacterium*, *Proteus*, *Staphylococcus*, *Enterococcus*, *Klebsiella*, *Micrococcus*, *Escherichia* and *Pseudomonas*. Gallic, vanillic, synapic and protocatechuic acids inhibit growth of Gram-positive bacteria, e.g., *Staphylococcus aureus* and *Staphylococcus epidermidis*, as well as Gram-negative bacteria, e.g., *Escherichia coli*, *Enterobacter cloacae* DG-6 and *Pseudomonas acidovorans* [44,45,46]. These acids exhibit a more effective destructive effect on cells of Gram-positive bacteria rather than Gram-negative bacteria. This results from the fact that cells of Gram-negative bacteria are equipped with an external capsule surrounding the cell wall, which hinders diffusion of hydrophobic compounds to the cell through the liposaccharide membrane. Such bioactive compounds as caffeic, ferulic and protocatechuic acids also inhibit growth of bacteria responsible for food poisonings, e.g., *Bacillus subtilis* and *Bacillus cereus*. Derivatives of phenolic acids exhibit also bactericidal properties against rods of *Yersinia enterocolitica*. Among them the compounds of o-coumaric acid are more effective than derivatives of m-coumaric acids, which may be bound both with the chemical structure of these phenolic acids and resistance of these bacteria [47].

Antimicrobial properties of phenolic acid extracts result from the antioxidant activity of these compounds, which depends e.g., on the amount of -OH groups and unsaturated bonds in the molecule. In plants phenolic acids are typically found in the bound form as esters and glycosides contained in lignins and hydrolysing tannins. Some hydroxycinnamic acids are found in ester combinations with carboxylic acids or with glucose. In plant tissues other combinations of phenolic acids are also found, e.g., flavonoids, fatty acids, sterols and polymers of cell walls. Phenolic acids may also be components of anthocyanins or flavones [48,49,50]. An example may be provided by caffeic acid, which are found in plants in the form of derivatives such as glycosides, amides and esters. Caffeic acid most frequently forms esters with quinic, α-hydroxydihydrocaffeic and tartaric acids, producing chlorogenic and rosmarinic. These derivatives exhibit greater antioxidant activity compared to free caffeic acid [35].

During the present study, the analyzes of phenolic compound extracts obtained from grains of naturally infected and inoculated fine grains were carried out. In the case of extracts from naturally infested grains, significantly lower levels of the tested bioactive compounds and significantly lower antioxidant activity were found. On the basis of the obtained results, it was found that the extracts of phenolic compounds differ quantitatively and qualitatively. It was found that the tested extracts consisted of phenolic acids, and the highest concentration of total phenolic compounds (TPC) in extracts from inoculated cereal grains: population rye (Agrikolo), fodder barley (KWS Harris) and malting barley (KWS Irina). On the other hand, the lowest concentration of TPC was found in grain extracts: common wheat (KWS Ozon, Milewo) and triticale (Borowik). Based on the conducted research, it was noticed that ferulic acid was the dominant compound in all the tested samples. Its concentration was the highest among all identified compounds, but it differed depending on the grain species from which the extract was obtained.

An important parameter responsible for the antioxidant properties and the resulting antimicrobial effects is antioxidant activity (ABTS^● +^). On the basis of this research, high antioxidant activity was found in all extracts from inoculated cereal grains, but the highest were those obtained from grain: common wheat (Astoria), fodder barley (Argento, KWS Harris), brewed barley (KWS Irina), triticale (Milkaro, Nagano) and common oats (Bingo). On the basis of the obtained results, it was found that the antioxidant activity of phenolic compound extracts derived from the grain of naturally infected cereals was at least two times lower than that of the inoculated cereal extracts.

This study shows that cereal grain contaminated with pathogens is rich in phenolic compounds and that extracts obtained from it are more antimicrobial active than the control. Therefore, they can become an element of biological preparations with antimicrobial activity used in organic or integrated agriculture.

## 5. Conclusions

The grain of commonly cultivated cereals in Poland is an important source of bioactive compounds of antioxidant nature. One of the most promising in this field is the group of phenolic acids. The ubiquity, availability, antioxidant and antimicrobial properties make polyphenols important naturally occurring components in plant cells. The concentration of these compounds depends primarily on the species and environmental factors. On the basis of the conducted research, it was found that a significantly higher concentration of phenolic compounds in the grain of cereals characterized by a high infestation with fungi of the *Fusarium* genus compared to the naturally infected grain. It was also noticed that the qualitative and quantitative profile of phenolic compound extracts is diversified and depends on the variety and use form of the grain.

On the basis of the next stage of the research, the obtained extracts of phenolic compounds were subjected to microbiological analysis. It was found then that the phenolic compound extracts derived from inoculated cereals had different properties of inhibiting the growth of the tested pathogens, while the extracts of the control cereals showed a complete lack of activity against the tested bacteria *Escherichia coli, Pseudomonas fluorescence, Micrococcus luteus* and *Proteus mirabilis*, as well as microscopic fungi of the genus *Fusarium* ssp.: *Fusarium culmorum, Fusarium graminearum, Fusarium langsethiae*. On the basis of the conducted research, bactericidal activity was found in the case of: 4 extracts against *M. luteus*, 14 extracts against *P. fluorescens*, 17 extracts against *E. coli* and 16 extracts against *P. mirabilis*. Moreover, it was noticed that 3 out of 24 tested grain extracts derived from inoculated cereals did not show any antibacterial activity. On the other hand, fungicidal properties were found in the case of: 17 extracts against *F. culmorum* (NIV), 16 extracts against *F. culmorum* (3AcDON), 12 extracts against *F. graminearum* (3AcDON), 12 other extracts against *F. graminearum* NIV and 19 extracts against *F. langsethiae.*

The extracts tested in this study are the basis for the creation of an antimicrobial preparation that can be used in organic farming.

## Figures and Tables

**Table 1:** Examples of plants extracts and their activity.

Plant	Type of Extract	Activity against Bacteria	Literature
Ginger (*Zingiber officinale*)	Ethanolic	*Staphylococcus aureus* *Escherichia coli* *Bacillus subtilis*	[7,8,9,10,11,12]
Dill (*Anethum graveolens*)	Ethanolic	*Staphylococcus aureus* *Escherichia coli*	
Lovage (*Levisticum officinale*)	Ethanolic	*Escherichia coli* *Staphylococcus aureus* *Pseudomonas aeruginosa* *Bacillus subtilis*	
Thyme (*Thymus vulgaris*)	Ethanolic	*Escherichia coli* *Staphylococcus aureus* *Bacillus subtilis*	
Rosemary (*Rosmarinus officinalis*)	Oil	*Salmonella bruneii* *Escherichia coli*	[10,13]
Oregano (*Origanum vulgare* L.)	Aqueous, Ethanolic, Essential oil	*Bacillus subtilis* *Staphylococcus ureus* *Micrococcus sp.* *Tetracoccus sp.* *Enterococcus faecalis* *Escherichia coli* *Proteus vulgaris* *Proteus mirabilis* *Klebsiella pneumonia* *Salmonella enteritidis*	[10,14,15]
Flowers of elderberry (*Sambucus nigra*)	Ethanolic	*Bacillus subtilis*, *Salmonella typhi*, *Staphylococcus aureus*, *Pseudomonas aeruginosa* *Klebsiella pneumoniae*	[16]
Flowers of elderberry (*Sambucus nigra*)	Methanolic	*Bacillus subtilis*, *Salmonella typhi*, *Staphylococcus aureus*, *Escherichia coli*, *Pseudomonas aeruginosa*	[17]
Fruits of large cranberry (*Vaccinium macrocarpon*)	Methanolic	*Salmonella ser. Enteritidis* *S. ser. Typhimurium* *Shigella sonnei* *Escherichia coli* *Klebsiella pneumonia* *Enterobacter aerogenes* *Proteus mirabilis* *Pseudomonas aeruginosa* *Staphylococcus aureus* *S. epidermidis* *Enterococcus faecalis* *Listeria monocytogenes* *Bacillus cereus*	[18]
*Adenanthera pavonina* L., *Moringa oleifera Lam.*, *Annona squamosa* L., *Hibiscus sabdariffa* L. *Eupotorium odortum* L.	Ethanolic	*Campylobacter jejuni*	[19,20]
Grape seeds Green tea	Aquo-alcoholic	*C. jejuni*	[21,22]
Sweet acacia (*Vachellia farnesiana*) Silver wormwood (*Artemisia ludoviciana*) Peppers (*Capsicum annuum*) Lemon grass (*Cymbopogon citratus*) Artichoke (*Cynara scolymus*) Mango (*Mangifera indica*) Basil (*Ocimum basilicum*) Prickly pear (*Opuntia ficus-indica*) Japanese plum (*Prunus salicina*) Red raspberry (*Rubus idaeus*)	Aquo-alcoholic	*C. jejuni* *C. coli*	[12,23,24,25]

**Table 2:** Selected small-grained cereals grown at the Dłoń Experimental Agricultural Station in the years 2017–2019.

Cereal Species	Form/Use Type	Cereal Cultivar	Breeder
Barley *Hordeum vulgare* L.	spring malting	Irina	KWS Lochow Polska sp. z o.o.
		Nokia	KWS Lochow Polska sp. z o.o.
	spring fodder	Argento	DANKO Hodowla Roślin sp. z o.o.
		Harris	KWS Lochow Polska sp. z o.o.
Hulless oat *Avena nuda* L.	spring	Amant	Hodowla Roślin Strzelce sp. z o.o. IHAR Group
		Siwek	Małopolska Hodowla Roślin sp. z o.o.
Common oat *Avena sativa* L.	spring	Bingo	Hodowla Roślin Strzelce sp. z o.o. IHAR Group
		Nawigator	Hodowla Roślin Strzelce sp. z o.o. IHAR Group
Wheat *Triticum durum* Desf.		SMH 87	Hodowla Roślin Strzelce sp. z o.o. IHAR Group
Wheat *Triticum aestivum* L.	winter	Astoria	Poznańska Hodowla Roślin sp. z o.o.
		Ozon	KWS Lochow Polska sp. z o.o.
	spring	Kandela	DANKO Hodowla Roślin sp. z o.o.
		Torka	Hodowla Roślin Strzelce sp. z o.o. IHAR Group
Triticale *Triticosecale* Wittm. ex A. Camus	spring	Dublet	DANKO Hodowla Roślin sp. z o.o.
		Milewo	Hodowla Roślin Strzelce sp. z o.o. IHAR Group
		Milkaro	Hodowla Roślin Strzelce sp. z o.o. IHAR Group
		Nagano	DANKO Hodowla Roślin sp. z o.o.
	winter	Palermo	DANKO Hodowla Roślin sp. z o.o.
		Borowik	Hodowla Roślin Strzelce sp. z o.o. IHAR Group
Rye *Secale cereale* L.	winter open pollinated	Agrikolo	Hodowla Roślin Strzelce sp. z o.o. IHAR Group
		Rostockie	Hodowla Roślin Strzelce sp. z o.o. IHAR Group
	winter hybrid	Dolaro	KWS Lochow Polska sp. z o.o..
		Tur	Hodowla Roślin Strzelce sp. z o.o. IHAR Group
		S74n05	DANKO Hodowla Roślin sp. z o.o.

**Table 3:** Concentrations [%] of extracts of phenolic acids coming from selected cereal genotypes grown in Poland and Minimum Lethal Concentration (MLC) of extracts against selected bacteria (ML, PF, EC, PM) and microscopic fungi (*Fusarium* spp).

	Cereal Species	Cereal Cultivar	Concentration of the Aqueous Extract of Phenolic Compounds [%]	Concentration of Phenolic Compounds in Extract [μg/g Extract]	Mean Major Compounds ± SD [μg/g extract] Inoculated with *Fusarium* spp.	MLC Extracts against Selected Pathogens [μg/g Extract]								
						Bacteria				Microscopic Fungi				
						ML	PF	EC	PM	KF350 FC NIV	KF846 FC 3AcDON	ZFR 29 FG 3AcDON	ZFR 119 FG NIV	8051 FL
WINTER INOCULATEDE	Wheat	Astoria	1.6	133.4	Ferulic	1.6 ± 0.05	0.6 ± 0.02	0.6 ± 0.03	0.6 ± 0.05	0.8 ± 0.03	0.8 ± 0.02	0.5 ± 0.03	0.25 ± 0.03	0.5 ± 0.04
	Wheat	KWS Ozon	2.54	72.6	Ferulic	2.54 ± 0.03	2.54 ± 0.06	0.95 ± 0.05	0.95 ± 0.04	2.54 ± 0.04	0.75 ± 0.03	0.5 ± 0.05	2.54 ± 0.04	1.27 ± 0.03
	Open pollinated rye	Rostockie	2.24	86.6	Ferulic, Gallic, Naringenin	2.24 ± 0.03	2.24 ± 0.04	0.84 ± 0.03	0.84 ± 0.04	2.24 ± 0.04	0.75 ± 0.04	0.25 ± 0.03	0.75 ± 0.05	0.25 ± 0.03
	Open pollinated rye	Agrikolo	1.62	138.27	Ferulic, Gallic, Naringenin	1.62 ± 0.05	0.61 ± 0.03	1.62 ± 0.04	1.62 ± 0.03	0.5 ± 0.05	0.5 ± 0.05	0.25 ± 0.04	1.62 ± 0.03	0.25 ± 0.04
	Hybrid rye	Tur	2.44	94.85	Ferulic, Naringenin	0.07 ± 0.005	0.91 ±0.03	2.44 ± 0.05	2.44 ± 0.04	0.25 ± 0.05	2.44 ± 0.04	1.22 ± 0.03	2.44 ± 0.04	1.22 ± 0.04
	Hybrid rye	Dolaro	2.68	84.81	Ferulic, Naringenin	2.68 ± 0.02	2.68 ± 0.05	2.68 ± 0.03	2.68 ± 0.03	2.68 ± 0.03	2.68 ± 0.03	2.68 ± 0.05	1.34 ± 0.04	1.34 ± 0.04
	Rye	S74n05	3.4	68.82	Ferulic, Sinapic	3.4 ± 0.04	3.4 ± 0.04	1.28 ± 0.03	1.28 ± 0.05	3.4 ± 0.05	3.4 ± 0.04	3.4 ± 0.06	3.4 ± 0.05	3.4 ± 0.05
	Triticale	Palermo	3.26	31.04	Ferulic, Sinapic	0.1 ± 0.01	1.22 ± 0.05	1.22 ± 0.04	1.22 ± 0.05	3.26 ± 0.04	3.26 ± 0.04	3.26 ± 0.04	3.26 ± 0.03	3.26 ± 0.04
	Triticale	Borowik	1.92	35.8	Ferulic, Sinapic	1.92 ± 0.4	1.92 ± 0.06	1.92 ± 0.05	1.92 ± 0.05	0.25 ± 0.05	0.5 ± 0.02	1.92 ± 0.05	0.96 ± 0.03	1.92 ±0.05
SPRING INOCULATEDE	Malting barley	Nokia	3.82	60.78	Ferulic, Naringenin	3.82 ± 0.05	1.43 ± 0.04	3.82 ±0.05	1.43 ± 0.02	1.91 ± 0.03	1.91 ± 0.02	3.82 ± 0.04	0.75 ± 0.05	0.75 ± 0.04
	Fodder barley	Argento	2.96	186.82	Ferulic, Quercitine, Naringenin	0.74 ± 0.03	0.98 ± 0.02	0.89 ± 0.03	0.74 ± 0.02	0.75 ± 0.04	0.75 ± 0.03	0.75 ± 0.05	0.75 ± 0.05	0.25 ± 0.03
	Triticale	Milkaro	2.66	68.19	Ferulic, Sinapic	2.66 ± 0.04	1.00 ± 0.05	1.00 ± 0.04	1.00 ± 0.03	0.75 ± 0.04	0.75 ± 0.03	2.66 ± 0.06	2.66 ± 0.03	2.66 ± 0.03
	Triticale	Dublet	3.5	34.74	Ferulic, Sinapic	3.5 ± 0.05	3.5 ± 0.05	1.31 ±0.03	1.31 ± 0.03	0.75 ± 0.03	3.5 ± 0.04	3.5 ± 0.04	0.25 ± 0.03	0.75 ± 0.04
	Fodder barley	KWS Harris	1.6	160.62	Ferulic, Quercitine, Naringenin	0.05 ± 0.001	0.6 ± 0.05	0.6 ± 0.04	1.6 ± 0.05	0.5 ± 0.03	0.8 ± 0.02	0.8 ± 0.03	1.6 ± 0.05	0.5 ± 0.03
	Hulless oat	Amant	3.46	59.63	Ferulic, Sinapic, Naringenin, Vitaxin	3.46 ± 0.04	1.3 ± 0.04	1.3 ± 0.03	1.3 ± 0.05	1.73 ± 0.04	3.46 ± 0.04	3.46 ± 0.03	3.46 ± 0.05	1.73 ± 0.03
	Wheat	Kandela	2.86	32.9	Ferulic	2.86 ± 0.05	2.86 ± 0.04	1.07 ± 0.03	1.07 ± 0.05	0.75 ± 0.03	0.75 ± 0.04	2.86 ± 0.05	0.75 ± 0.04	0.25 ± 0.02
	Malting barley	KWS Irina	1.9	72.52	Ferulic, Quercitine, Naringenin	1.9 ± 0.02	0.71 ± 0.03	0.71 ± 0.02	0.71 ± 0.04	1.9 ± 0.04	0.95 ± 0.05	1.9 ± 0.04	1.9 ± 0.04	0.5 ± 0.04
	Hulless oat	Siwek	3.66	53.82	Ferulic, Sinapic, Naringenin, Vitaxin	3.66 ± 0.04	3.66 ± 0.04	1.37 ± 0.04	1.37 ± 0.03	1.83 ± 0.03	3.66 ± 0.05	3.66 ± 0.04	0.25 ± 0.03	1.83 ± 0.03
	Triticale	Milewo	3.88	63.2	Ferulic	3.88 ± 0.05	1.46 ± 0.04	1.46 ± 0.04	1.46 ± 0.04	0.75 ± 0.03	0.75 ± 0.05	0.75 ± 0.03	3.88 ± 0.03	3.88 ± 0.05
	Durum wheat	SMH 87	2.96	12.5	Ferulic	2.96 ± 0.04	2.96 ± 0.04	2.96 ± 0.05	2.96 ± 0.04	0.75 ± 0.05	0.75 ± 0.03	0.75 ± 0.05	2.96 ± 0.04	0.75 ± 0.04
	Wheat	Torka	2.72	69.23	Ferulic, Sinapic	2.72 ± 0.05	1.02 ± 0.05	1.02 ± 0.05	1.02 ± 0.03	0.25 ± 0.04	2.72 ± 0.03	2.72 ± 0.05	2.72 ± 0.05	0.75 ± 0.03
	Triticale	Nagano	1.94	94.63	Ferulic, Sinapic	1.94 ± 0.03	0.73 ± 0.03	0.73 ± 0.02	0.73 ± 0.03	1.94 ± 0.04	0.5 ± 0.04	0.25 ± 0.03	0.97 ± 0.04	0.25 ± 0.03
	Hulled oat	Nawigator	2.94	125.6	Ferulic, Sinapic, t-Cinnamic, Naringenin, Vitaxin	2.94 ± 0.04	1.1 ± 0.02	2.94 ± 0.04	2.94 ± 0.05	0.75 ± 0.04	0.75 ± 0.04	0.75 ± 0.04	0.75 ± 0.04	0.75 ± 0.04
	Hulled oat	Bingo	2.38	119.63	Ferulic, Sinapic, t-Cinnamic, Naringenin, Vitaxin	2.38 ± 0.03	2.38 ± 0.04	0.89 ± 0.04	2.38 ± 0.05	0.5 ± 0.03	0.75 ± 0.05	0.75 ± 0.05	0.75 ± 0.05	1.19 ± 0.04

**Table 4:** Concentrations [%] of extracts of phenolic acids from selected genotypes of cereals grown in Poland and Minimum Inhibitory Concentration (MIC) of extracts against selected bacteria (ML, PF, EC, PM) and microscopic fungi (*Fusarium* spp.).

	Cereal Species	Cereal Cultivar	Concentration of the Aqueous Extract of Phenolic Compound [%]	Concentration of Phenolic Compounds in Extract [μg/g Extract]	Mean Major Compounds ± SD [μg/g Extract] Inoculated with *Fusarium* spp.	MIC of Extracts against Selected Pathogens [μg/g Extract]								
						Bacteria				Microscopic Fungi				
						ML	PF	EC	PM	KF350 FC NIV	KF846 FC 3AcDON	ZFR 29 FG3AcDON	ZFR 119 FG NIV	FL 8051
WINTER INOCULATED	Wheat	Astoria	1.6	133.4	Ferulic	+	0.48 ± 0.03	0.48 ± 0.01	0.48 ± 0.02	0.64 ± 0.01	0.64 ± 0.02	0.40 ± 0.01	0.20 ± 0.01	0.40 ± 0.02
	Wheat	KWS Ozon	2.54	72.6	Ferulic	+	+	0.76 ± 0.02	0.76 ± 0.02	+	0.60 ± 0.02	0.40 ± 0.01	+	1.02 ± 0.03
	Open pollinated rye	Rostockie	2.24	86.6	Ferulic, Gallic, Naringenin	+	+	0.67 ± 0.01	0.67 ± 0.02	+	0.60 ± 0.02	0.20 ± 0.01	0.60 ± 0.02	0.20 ± 0.01
	Open pollinated rye	Agrikolo	1.62	138.27	Ferulic, Gallic, Naringenin	+	0.49 ± 0.02	+	+	0.40 ± 0.01	0.40 ± 0.01	0.20 ± 0.01	+	0.20 ± 0.02
	Hybrid rye	Tur	2.44	94.85	Ferulic, Naringenin	0.06 ± 0.001	0.73 ± 0.03	+	+	0.20 ± 0.02	+	0.98 ± 0.03	+	0.98 ± 0.03
	Hybrid rye	KWS Dolaro	2.68	84.81	Ferulic, Naringenin	+	+	+	+	+	+	+	1.07 ± 0.03	1.07 ± 0.03
	Rye	S74n05	3.4	68.82	Ferulic, Sinapic	+	+	1.02 ± 0.02	1.02 ± 0.01	+	+	+	+	+
	Triticale	Palermo	3.26	31.04	Ferulic, Sinapic	0.08 ± 0.005	0.98 ± 0.02	0.98 ± 0.02	0.98	+	+	+	+	+
	Triticale	Borowik	1.92	35.8	Ferulic, Sinapic	+	+	+	+	0.20 ± 0.02	0.40 ± 0.02	+	0.77 ± 0.02	+
SPRING INOCULATED	Malting barley	Nokia	3.82	60.78	Ferulic, Naringenin	+	1.14 ± 0.03	+	1.14 ± 0.03	1.53 ± 0.03	1.53 ± 0.02	+	0.60 ± 0.02	0.60 ± 0.02
	Fodder barley	Argento	2.96	186.82	Ferulic, Quercitine, Naringenin	0.59 ± 0.03	0.78 ± 0.02	0.71 ± 0.01	0.59 ± 0.01	0.60 ± 0.01	0.60 ± 0.01	0.60 ± 0.02	0.60 ± 0.02	0.20 ± 0.02
	Triticale	Milkaro	2.66	68.19	Ferulic, Sinapic	+	0.80 ± 0.02	0.80 ± 0.02	0.80 ± 0.02	0.60 ± 0.01	0.60 ± 0.02	+	+	+
	Triticale	Dublet	3.5	34.74	Ferulic, Sinapic	+	+	1.05 ± 0.03	1.05 ± 0.03	0.60 ± 0.01	+	+	0.20 ± 0.01	0.60 ± 0.02
	Fodder barley	KWS Harris	1.6	160.62	Ferulic, Quercitine, Naringenin	0.04 ± 0.005	0.48 ± 0.01	0.48 ± 0.01	+	0.40 ± 0.01	0.64 ± 0.02	0.64 ± 0.02	+	0.40 ± 0.01
	Hulless oat	Amant	3.46	59.63	Ferulic, Sinapic, Naringenin, Vitaxin	+	1.04 ± 0.02	1.04 ± 0.03	1.04 ± 0.03	1.38 ± 0.03	+	+	+	1.38 ± 0.03
	Wheat	Kandela	2.86	32.9	Ferulic	+	+	0.86 ± 0.02	0.86 ± 0.01	0.60 ± 0.02	0.60 ± 0.02	+	0.60 ± 0.02	0.20 ± 0.01
	Malting barley	KWS Irina	1.9	72.52	Ferulic, Quercitine, Naringenin	+	0.57 ± 0.01	0.57 ± 0.01	0.57 ± 0.01	+	0.76 ± 0.2	+	+	0.40 ± 0.02
	Hulless oat	Siwek	3.66	53.82	Ferulic, Sinapic, Naringenin, Vitaxin	+	+	1.10 ± 0.02	1.10 ± 0.03	1.46 ± 0.03	+	+	0.20 ± 0.01	1.46 ± 0.03
	Wheat	Milewo	3.88	63.2	Ferulic	+	1.17 ± 0.02	1.17 ± 0.02	1.17 ± 0.03	0.60 ± 0.01	0.60 ± 0.01	0.60 ± 0.02	+	+
	Durum wheat	SMH 87	2.96	12.5	Ferulic	+	+	+	+	0.60 ± 0.01	0.60 ± 0.01	0.60 ± 0.02	+	0.60 ± 0.02
	Wheat	Torka	2.72	69.23	Ferulic, Sinapic	+	0.82 ± 0.01	0.82 ± 0.01	0.82 ± 0.02	0.20 ± 0.01	+	+	+	0.60 ± 0.02
	Triticale	Nagano	1.94	94.63	Ferulic, Sinapic	+	0.58 ± 0.01	0.58 ± 0.01	0.58 ± 0.02	+	0.40 ± 0.01	0.20 ± 0.01	0.78 ± 0.03	0.20 ± 0.02
	Hulled oat	Nawigator	2.94	125.6	Ferulic, Sinapic, t-Cinnamic, Naringenin, Vitaxin	+	0.88 ± 0.02	+	+	0.60 ± 0.01	0.60 ± 0.02	0.60 ± 0.01	0.60 ± 0.02	0.60 ± 0.01
	Hulled oat	Bingo	2.38	119.63	Ferulic, Sinapic, t-Cinnamic, Naringenin, Vitaxin	+	+	0.71 ± 0.03	+	0.40 ± 0.01	0.60 ± 0.02	0.60 ± 0.02	0.60 ± 0.02	0.95 ± 0.3

+ growth of bacteria and microscopic fungi.

**Table 5:** Concentration of bee phenolic acids in extracts obtained from naturally infected cereal grains.

Cereal Species		Cereal Cultivar	K.Ga	K.2.5-Hb	K.4-Hb	K.tC	K.Ka	K.Syr	K.pK	K.Chl	K.Pr	K.Sy	K.Fe
WINTER INOCULATED	Open pollinated rye	Agrokol	2.1 ± 0.01	1.6 ± 0.02	1.5 ± 0.02	0.5 ± 0.01	3.4 ± 0.03	8.0 ± 0.11	1.3 ± 0.02	2.0 ± 0.02	1.0 ± 0.02	1.6 ± 0.03	69.1 ± 0.74
	Wheat	Astoria	0.5 ± 0.01	0.5 ± 0.01	0.5 ± 0.01	0.1 ± 0.01	0.5 ± 0.01	8.7 ± 0.09	0.8 ± 0.02	1.0 ± 0.01	0.6 ± 0.01	0.5 ± 0.01	20.0 ± 0.32
	Triticale	Borowik	2.3 ± 0.02	0.3 ± 0.01	0.4 ± 0.01	0.0 ± 0.01	0.2 ± 0.01	5.6 ± 0.07	0.1 ± 0.01	0.3 ± 0.01	0.1 ± 0.01	0.6 ± 0.01	26.1 ± 0.35
	Hybrid rye	Doloro	4.6 ± 0.03	0.6 ± 0.01	0.4 ± 0.01	0.2 ± 0.01	0.3 ± 0.01	7.4 ± 0.07	0.1 ± 0.01	0.5 ± 0.01	0.4 ± 0.01	1.3 ± 0.02	51.5 ± 0.62
	Wheat	Ozon	2.2 ± 0.02	1.9 ± 0.02	3.0 ± 0.03	0.5 ± 0.01	1.2 ± 0.02	5.1 ± 0.06	0.4 ± 0.01	0.9 ± 0.01	0.2 ± 0.01	2.8 ± 0.02	79.7 ± 0.87
	Triticale	Palermo	1.1 ± 0.01	0.9 ± 0.01	0.4 ± 0.01	0.3 ± 0.01	0.7 ± 0.01	16.8 ± 0.17	0.2 ± 0.01	0.6 ± 0.01	0.1 ± 0.01	1.4 ± 0.02	45.2 ± 0.51
	Open pollinated rye	Roztockie	0.5 ± 0.01	1.1 ± 0.02	0.4 ± 0.01	0.1 ± 0.01	0.5 ± 0.01	6.4 ± 0.06	0.1 ± 0.01	0.6 ± 0.01	0.1 ± 0.01	5.4 ± 0.4	30.6 ± 0.39
	Hybrid rye	S74n05	0.8 ± 0.01	0.4 ± 0.01	1.0 ± 0.01	0.1 ± 0.01	0.4 ± 0.01	21.1 ± 0.19	3.0 ± 0.02	0.2 ± 0.01	0.2 ± 0.01	13.3 ± 0.11	27.8 ± 0.38
	Hybrid rye	Tur f1	0.5 ± 0.01	0.4 ± 0.01	0.6 ± 0.01	0.1 ± 0.01	0.2 ± 0.01	19.7 ± 0.21	1.9 ± 0.02	0.2 ± 0.01	0.1 ± 0.01	8.6 ± 0.07	17.4 ± 0.28
SPRING INOCULATED	Hulless oat	Amant	0.5 ± 0.01	1.3 ± 0.02	0.4 ± 0.01	0.3 ± 0.01	0.5 ± 0.01	56.1 ± 0.43	0.8 ± 0.01	0.4 ± 0.01	0.8 ± 0.01	0.2 ± 0.01	32.5 ± 0.39
	Fodder barley	Argento	0.7 ± 0.01	1.8 ± 0.02	0.6 ± 0.01	0.5 ± 0.01	0.7 ± 0.01	52.1 ± 0.41	1.1 ± 0.02	0.6 ± 0.01	1.2 ± 0.01	0.2 ± 0.01	40.1 ± 0.51
	Hulled oat	Bingo	0.7 ± 0.01	0.3 ± 0.01	2.8 ± 0.03	0.1 ± 0.01	0.1 ± 0.01	42.5 ± 0.38	3.9 ± 0.03	0.1 ± 0.01	0.1 ± 0.01	12.0 ± 0.09	40.8 ± 0.59
	Triticale	Dublet	1.5 ± 0.02	1.3 ± 0.02	3.1 ± 0.03	0.2 ± 0.01	0.6 ± 0.01	30.4 ± 0.35	3.4 ± 0.03	0.5 ± 0.01	0.5 ± 0.01	8.9 ± 0.07	61.7 ± 0.98
	Wheat	Durum Smh 87	0.1 ± 0.01	0.5 ± 0.01	0.0 ± 0.01	0.0 ± 0.01	0.1 ± 0.01	50.8 ± 0.42	0.2 ± 0.01	0.0 ± 0.01	0.3 ± 0.01	0.0 ± 0.01	8.0 ± 0.16
	Fodder barley	Harris	0.5 ± 0.01	0.3 ± 0.01	0.1 ± 0.01	0.0 ± 0.01	1.1 ± 0.01	12.5 ± 0.11	0.8 ± 0.01	0.4 ± 0.01	1.1 ± 0.01	3.3 ± 0.02	21.2 ± 0.33
	Malting barley	Irina	1.3 ± 0.02	1.6 ± 0.02	0.6 ± 0.01	0.1 ± 0.01	1.8 ± 0.02	6.0 ± 0.05	1.4 ± 0.02	1.4 ± 0.02	0.2 ± 0.01	0.3 ± 0.01	28.2 ± 0.36
	Wheat	Kandela	0.8 ± 0.01	2.0 ± 0.03	0.7 ± 0.01	0.2 ± 0.01	0.7 ± 0.01	59.8 ± 0.51	1.3 ± 0.02	0.6 ± 0.01	1.3 ± 0.02	0.2 ± 0.01	38.9 ± 0.45
	Wheat	Milewo	0.7 ± 0.01	0.5 ± 0.01	0.7 ± 0.01	0.0 ± 0.01	1.2 ± 0.01	21.2 ± 0.19	0.5 ± 0.01	0.3 ± 0.01	0.4 ± 0.01	3.0 ± 0.03	30.9 ± 0.41
	Triticale	Milkaro	0.8 ± 0.01	1.0 ± 0.02	0.4 ± 0.01	0.1 ± 0.01	0.7 ± 0.01	13.3 ± 0.15	2.0 ± 0.02	0.4 ± 0.01	0.5 ± 0.01	0.9 ± 0.02	27.5 ± 0.39
	Triticale	Nagano	0.5 ± 0.01	1.6 ± 0.02	0.8 ± 0.01	0.0 ± 0.01	0.4 ± 0.01	19.4 ± 0.14	0.1 ± 0.01	0.3 ± 0.01	0.4 ± 0.01	0.1 ± 0.01	27.5 ± 0.39
	Hulled oat	Nawigator	0.9 ± 0.01	1.2 ± 0.02	0.5 ± 0.01	0.1 ± 0.01	1.1 ± 0.01	9.9 ± 0.12	1.1 ± 0.01	0.9 ± 0.01	0.2 ± 0.01	0.3 ± 0.01	18.9 ± 0.30
	Malting barley	Nokia	0.6 ± 0.01	0.1 ± 0.01	3.7 ± 0.04	0.2 ± 0.01	0.2 ± 0.01	44.6 ± 0.36	5.3 ± 0.03	0.1 ± 0.01	0.0 ± 0.01	17.0 ± 0.15	46.6 ± 0.48
	Hulless oat	Siwek	0.5 ± 0.01	0.2 ± 0.01	0.4 ± 0.01	3.9 ± 0.04	0.5 ± 0.01	20.9 ± 0.18	0.5 ± 0.01	0.4 ± 0.01	0.5 ± 0.01	4.9 ± 0.07	38.0 ± 0.49
	Wheat	Torka	0.4 ± 0.01	0.2 ± 0.01	0.2 ± 0.01	2.5 ± 0.03	0.7 ± 0.01	17.2 ± 0.15	0.3 ± 0.01	0.2 ± 0.01	0.7 ± 0.01	2.6 ± 0.04	24.2 ± 0.27

K.Ga—gallic acid; K.2.5-Hb—2.5 hydroxybenzoic acid; K.4-Hb—4-hydroxybenzoic acid; K.tC—t-cinnamic acid; K.Ka—caffeic acid; K.Syr—syrynic acid; K.pK—p-coumaric acid; K.Chl—chlorogenic acid; K.Pr—protocatechinic acid; K.Sy—synapic acid; K.Fe—ferulic acid.

**Table 6:** Concentration of individual phenolic acids in extracts obtained from inoculated cereal grains.

	Cereal Species	Cereal Cultivar	K.Ga	K.2.5-Hb	K.4-Hb	K.tC	K.Ka	K.Syr	K.pK	K.Chl	K.Pr	K.Sy	K.Fe
WINTER INOCULATED	Open pollinated rye	Agrokol	61.7 ± 0.02	8.1 ± 0.02	5.9 ± 0.03	2.2 ± 0.24	3.6 ± 0.23	4.6 ± 0.15	1.6 ± 0.02	7.3 ± 0.02	4.9 ± 0.02	17 ± 0.24	690.6 ± 2.54
	Wheat	Astoria	15.2 ± 0.02	11.6 ± 0.08	10.9 ± 0.02	3.9 ± 0.08	25.1 ± 0.02	5 ± 0.08	9.4 ± 0.02	14.3 ± 0.02	7.3 ± 0.24	11.6 ± 0.02	508.1 ± 2.84
	Triticale	Borowik	5 ± 0.08	3.5 ± 0.02	5.8 ± 0.02	0.5 ± 0.02	1.7 ± 0.02	10.3 ± 0.15	18.5 ± 0.15	1.7 ± 0.24	1.3 ± 0.14	83.2 ± 0.02	168 ± 3.14
	Hybrid rye	Doloro	10.3 ± 0.08	8.4 ± 0.02	3.9 ± 0.25	2.5 ± 0.02	6.5 ± 0.08	6.3 ± 0.02	1.8 ± 0.2	5.4 ± 0.02	1 ± 0.02	12.5 ± 0.24	411.5 ± 3.04
	Wheat	Ozon	4 ± 0.15	4.4 ± 0.24	4.1 ± 0.02	1 ± 0.02	4.4 ± 0.15	3.4 ± 0.02	6.8 ± 0.02	9 ± 0.02	4.9 ± 0.02	4.1 ± 0.15	174.9 ± 1.54
	Triticale	Palermo	3.1 ± 0.15	1.6 ± 0.08	4 ± 0.02	0.4 ± 0.05	1.7 ± 0.02	6.5 ± 0.08	12.6 ± 0.2	1 ± 0.13	1 ± 0.07	55.1 ± 0.02	115.4 ± 2.96
	Open pollinated rye	Roztockie	37 ± 0.02	5 ± 0.02	6.1 ± 0.02	0.6 ± 0.05	2.4 ± 0.02	2.5 ± 0.02	1.1 ± 0.02	4.2 ± 0.15	0.9 ± 0.02	9.9 ± 0.24	416.2 ± 2.75
	Hybrid rye	S74n05	3.6 ± 0.08	8.5 ± 0.15	2.7 ± 0.08	0.7 ± 0.02	3.7 ± 0.02	1.9 ± 0.14	0.8 ± 0.08	4.5 ± 0.02	0.5 ± 0.02	40.4 ± 0.02	231 ± 3.08
	Hybrid rye	Tur f1	10.3 ± 0.02	8.7 ± 0.02	14.1 ± 0.24	2.2 ± 0.02	5.6 ± 0.22	2.1 ± 0.17	1.7 ± 0.02	4.2 ± 0.02	0.9 ± 0.07	12.8 ± 0.02	371.3 ± 3.13
SPRING INOCULATED	Hulless oat	Amant	5.6 ± 0.02	2.7 ± 0.08	1.4 ± 0.02	0.4 ± 0.02	11.2 ± 0.34	3.6 ± 0.02	7.9 ± 0.02	4.4 ± 0.02	11.2 ± 0.14	34.1 ± 0.34	218.3 ± 2.54
	Fodder barley	Argento	6.1 ± 0.24	15.9 ± 0.02	5.3 ± 0.02	4.6 ± 0.08	6 ± 0.08	17.6 ± 0.02	10 ± 0.08	5.3 ± 0.14	10.6 ± 0.02	1.7 ± 0.08	356.1 ± 2.55
	Hulled oat	Bingo	6.9 ± 0.02	4.6 ± 0.08	4.2 ± 0.08	47.2 ± 0.02	13.5 ± 0.15	7.2 ± 0.21	4.7 ± 0.15	3.4 ± 0.15	13.5 ± 0.02	48.1 ± 0.02	455.8 ± 2.85
	Triticale	Dublet	5.8 ± 0.08	5.1 ± 0.02	12 ± 0.15	0.9 ± 0.02	2.4 ± 0.02	8.7 ± 0.15	13.1 ± 0.02	2 ± 0.02	1.8 ± 0.09	34.3 ± 0.15	238.2 ± 3.54
	Wheat	Durum Smh 87	4.6 ± 0.02	13.9 ± 0.24	7.5 ± 0.02	0.4 ± 0.04	3.8 ± 0.02	6.5 ± 0.02	0.7 ± 0.04	2.4 ± 0.02	3.1 ± 0.02	1 ± 0.15	244.4 ± 3.84
	Fodder barley	Harris	4 ± 0.24	27.7 ± 0.02	2.2 ± 0.02	1.6 ± 0.04	3.8 ± 0.14	31.8 ± 0.34	13.4 ± 0.14	2 ± 0.15	14.9 ± 0.15	2.1 ± 0.24	454.3 ± 2.93
	Malting barley	Irina	10.4 ± 0.02	28.4 ± 0.08	9.1 ± 0.08	2.2 ± 0.02	10.3 ± 0.08	31.5 ± 0.44	17.5 ± 0.02	8.9 ± 0.08	18.7 ± 0.15	2.4 ± 0.23	538.8 ± 3.54
	Wheat	Kandela	9 ± 0.02	11.2 ± 0.15	4.1 ± 0.24	0.8 ± 0.02	12.5 ± 0.08	2.1 ± 0.14	9. 6± 0.02	9.7 ± 0.08	1.5 ± 0.15	1.9 ± 0.08	201.2 ± 1.74
	Wheat	Milewo	4.4 ± 0.02	6 ± 0.24	2.4 ± 0.02	0.6 ± 0.03	4.3 ± 0.02	3.4 ± 0.15	11.8 ± 0.02	2.5 ± 0.15	2.6± 0.02	5.5 ± 0.15	161.2 ± 2.56
	Triticale	Milkaro	5.6 ± 0.08	2.6 ± 0.02	23.2 ± 0.02	0.5 ± 0.02	0.8 ± 0.02	16 ± 0.15	32.2 ± 0.08	0.8	1.1 ± 0.02	97.8 ± 0.02	333.7 ± 2.55
	Triticale	Nagano	5.6 ± 0.02	0.7± 0.02	35 ± 0.02	2.2 ± 0.14	1.5 ± 0.13	23 ± 0.28	50.1 ± 0.45	1.3 ± 0.02	0.1 ± 0.04	159.4 ± 0.84	436.9 ± 2.34
	Hulled oat	Nawigator	4.9 ± 0.24	2.1 ± 0.02	3.7 ± 0.08	35.8 ± 0.08	4.7 ± 0.02	7.1 ± 0.24	4.3 ± 0.02	4 ± 0.02	4.2 ± 0.02	45.3 ± 0.02	349.1 ± 2.15
	Malting barley	Nokia	4.8 ± 0.02	12.2 ± 0.15	4.3 ± 0.24	3.3 ± 0.02	4.8 ± 0.15	14.7 ± 0.15	7.7 ± 0.02	4.2 ± 0.08	8.2 ± 0.02	1.5 ± 0.08	315.2 ± 3.54
	Hulless oat	Siwek	7.4 ± 0.02	5.4 ± 0.02	7 ± 0.02	0.4 ± 0.03	12.2 ± 0.02	5.8 ± 0.08	5 ± 0.08	3.2 ± 0.08	3.7 ± 0.15	30.8 ± 0.02	312.7 ± 3.57
	Wheat	Torka	11.5 ± 0.02	15.1 ± 0.02	6.3 ± 0.02	1.2 ± 0.02	14.4 ± 0.15	3.6 ± 0.08	14.1 ± 0.15	11.3 ± 0.02	3.1 ± 0.12	4 ± 0.24	248.5 ± 2.59

K.Ga—gallic acid; K.2.5-Hb—2.5 hydroxybenzoic acid; K.4-Hb—4-hydroxybenzoic acid; K.tC—t-cinnamic acid; K.Ka—caffeic acid; K.Syr—syrynic acid; K.pK—p-coumaric acid; K.Chl—chlorogenic acid; K.Pr—protocatechinic acid; K.Sy—synapic acid; K.Fe—ferulic acid.
